# Research on digital twin modeling and monitoring technology for smoke alarm calibration system

**DOI:** 10.1038/s41598-023-46761-1

**Published:** 2023-11-07

**Authors:** Wu Min, Ying Wenfeng

**Affiliations:** 1 School of Mechanical and Electrical Engineering, Ningbo Polytechnic, No.1069, Xindalu, Beilunqu, Ningbo, 315800 Zhejiang China; 2 Ningbo Weizmat Electronics Co., Ltd., Ningbo, 315000 China

**Keywords:** Electrical and electronic engineering, Computer science

## Abstract

Aiming at the problems of backward calibration method of smoke alarm, low production efficiency and difficult real-time monitoring, a digital twin system modeling and monitoring method of smoke alarm calibration is proposed. First, through the analysis of smoke alarm calibration requirements, the overall framework design of the digital twin calibration system for smoke alarm is proposed, and then the twin model of smoke box is constructed by running the digital twin five-dimensional model. The physical smoke box, geometric model, physical model, rule model and behavior model are introduced in detail; Then the communication system architecture, data acquisition and data mapping are used to construct the twin data model; Finally, the feasibility was verified by the calibration system of an enterprise. Through the system, the qualified rate of smoke alarm calibration was increased from 98 to 99.6%, and the repair rate of defective products was reduced by 1.6%.

## Introduction

Smoke alarm, which monitors the concentration of smoke to prevent fire. So far, smoke alarm is still the most widely used fire detection and alarm product in the market^1^. The smoke alarm can detect the smoke generated in the early stage of a fire and send an alarm signal when the smoke concentration reaches the alarm threshold^2^. Sensitivity is an important technical index of smoke alarm. The sensitivity of smoke alarm directly determines the quality of smoke alarm. When the manufacturer of smoke alarm produces smoke alarm, it is necessary to detect the sensitivity of each smoke alarm. The national standard, UL of the United States and en of Europe stipulate the fire sensitivity test respectively. Smoke alarm products need to pass the test standard of fire sensitivity. Due to the individual differences between the photoelectric transmitting and receiving pairs and the optical maze, the smoke alarm products need to be calibrated on a single chip in order to ensure that the sensitivity meets the specification^3^. The traditional calibration method uses manual methods to pick and place products and observe the calibration instrument data with the naked eye. This detection method has poor operability and low detection efficiency. With the increasing demand for smoke alarms, their quantity and types continue to increase. The smoke alarm calibration workshop will generate massive data in the production and operation process. How to use these data to establish a multi domain fusion data model and achieve an efficient application mode of real-time interaction between the data model and physical entities is a problem to be solved in the current smoke alarm production process. Digital twin technology provides the possibility to solve this problem.

Digital twin establishes a multi-dimensional, multi-dimensional, multi-disciplinary and multi physical dynamic virtual model of physical entities in a digital way to simulate and characterize the properties, behaviors and rules of physical entities in the real environment^4^. Digital twin technology is a technology that mirrors the physical entity space to the virtual world by means of computer technology through sensors or other forms of monitoring technology.

Digital twin technology has been applied and studied in the fields of satellite/space communication networks, ships, vehicles, power plants, aircraft, complex electromechanical equipment, three-dimensional warehouses, medical treatment, manufacturing workshops, smart cities, intelligent appliances, intelligent logistics, buildings, remote monitoring, and human health management^5^. Martins et al. discussed the Digital Twin is a holistic approach for supporting and controlling systems for in-orbit Assembly, Integration, and Test processes, as well as satellite operations, by establishing a bidirectional link to its physical counterpart^6^; Anyfantis et al. proposed two digital twin condition monitoring of ship hull structures systems, which obtain sensor readings from the structure as input sensor readings from the structure and provide the damage locus as an output^7^; Zhang, et al. introduce the digital twin of a real-world EV by modeling the mobility based on a time series behaviors of EVs to evaluate the charging algorithm and pile arrangement policy^8^; Deon et al. based on digital twin models for thermoelectric generation engines and their subsystems associated with models of machine learning for predictive maintenance, allowing the classification of failures in the generating units of the plant. The models represent the mechanical, thermal, and electrical conditions and parameters of each piece of equipment under normal operating conditions, and the tool generates alerts when deviations from the base model occur^9^; Kosova et al. proposed a novel digital twin-based health monitoring system for aircraft hydraulic systems to enable diagnostics of system failures early in the design cycle using machine learning (ML) methods^10^; Leung et al. Proposed a digital twin-based inbound synchronization framework to streamline the operations of a PI-hub in a hyperconnected city logistics system^11^; Lal et al. To develop and verify a digital twin model of critically ill patient using the causal artificial intelligence approach to predict the response to specific treatment during the first 24 h of sepsis^12^; Zhang et al. proposed a digital twin-driven carbon emission prediction and low-carbon control of intelligent manufacturing job-shop ,realize the carbon emission reduction in intelligent manufacturing workshop^13^; Zhongjun et al. create digital twins for historical buildings, to monitor historical buildings, predict energy consumption, and independently control energy consumption equipment, in order to achieve a balance between energy efficiency, building energy efficiency and human comfort^14^.

As for the research on the calibration of smoke alarm, only two literatures were retrieved with the keywords of "smoke alarm calibration", "smoke sensor calibration" and "smoke detector calibration", and the literature research was the elaboration of the calibration method of centralized alarm; There are only two literatures with the keyword of "alarm smoke box", which are about the performance research of smoke box; However, there is no relevant research on the digital twin of smoke alarm calibration. This paper studies the modeling method of smoke alarm calibrated smoke box by taking the digital twin modeling of smoke alarm calibrated smoke box as the breakthrough point.

## Working mechanism of smoke alarm calibration system

### Structural composition of smoke box system

**Figure 1:**
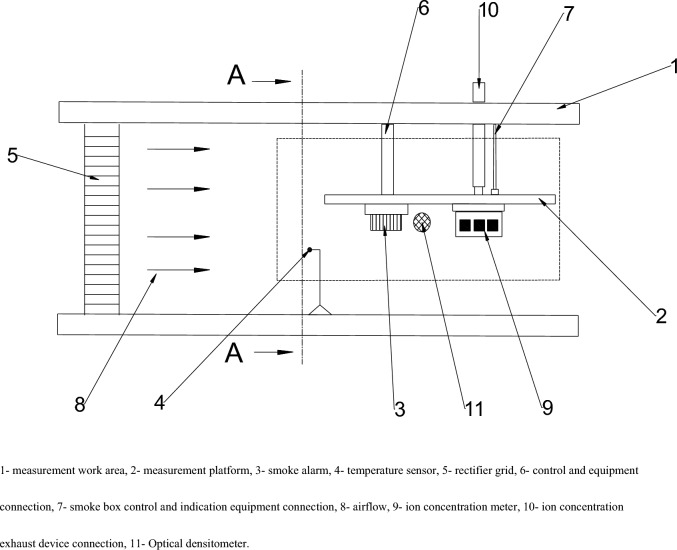
Schematic diagram of smoke alarm, smoke box and calibration measurement area.

The threshold calibration of smoke alarm shall be carried out in the smoke box. The structure of the smoke box system mainly includes: measurement work area, measurement platform, temperature and humidity sensor, flow rate sensor, rectifier grid, control and equipment connection, smoke box control and indication equipment connection, smoke generator, ion concentration meter, ion concentration exhaust device connection, optical densitometer, etc. Figure 1 shows the schematic diagram of smoke alarm, smoke box and calibration measurement area.

### Mathematical model of response threshold measured by optical method

The response threshold value of the smoke alarm, that is, the smoke concentration when the smoke alarm alarms is represented by the dimming coefficient m value, is measured by the optical densitometer, which measures the smoke density based on the principle that the light radiation energy attenuates exponentially after the beam is affected by the smoke particles^15^. The dimming coefficient model is expressed in digital language as (1):

1 $$m=(10/d){\log}\left({P}_{0}/P\right)$$

where: *m* is the dimming system, dBm^−1^; *d* is the optical measurement length of the experimental smoke, m; *P*_*0*_ is radiation power received when smokeless, W;* P* is radiation power received with smoke, W.

## Mathematical twin smoke box modeling and overall scheme design of monitoring

### Demand analysis

**Figure 2:**
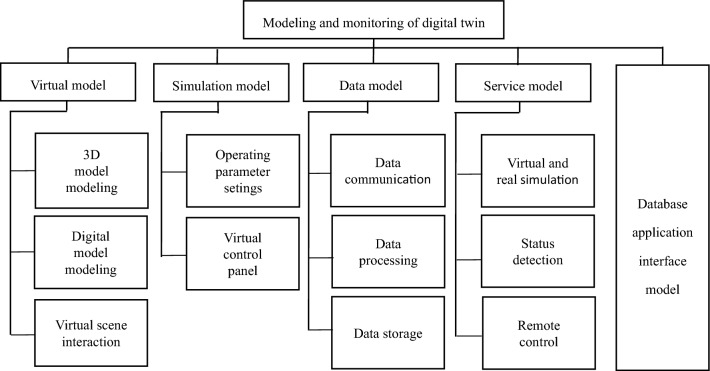
Mathematical twin smoke box modeling and monitoring requirements framework.

Smoke box modeling and monitoring requirements are divided into virtual model, simulation model, data model, service model and database application interface. As shown in Fig. 2.

Virtual model: build a virtual model that can be exchanged with physical entities. The virtual model is consistent with the production of physical equipment through mapping, including shape, position, action and mutual relationship. At the same time, the virtual model can also realize the monitoring, analysis and diagnosis of the production process.

Simulation model: mainly used to realize the simulation of cigarette box production and operation. By setting the parameters such as wind speed, temperature and humidity, and shielding rate of the smoke box, combined with the virtual model, the calibration motion simulation of the smoke box can be realized; The virtual control panel is established based on the physical smoke box. The user can debug the motion simulation of the virtual smoke box and send instructions to the physical smoke box for real-time remote control, so as to improve the practicability, interactivity and efficiency of the monitoring scheme.

Data model: collect the data during the operation of the smoke box in real time, integrate and clean the data, and store the effective data in the database. It can also run the collected real-time data to control the virtual model motion simulation.

Service model: realize virtual simulation, virtual real synchronous simulation, production status monitoring, remote control and other application services based on the model.

Database application interface: it is mainly used to connect the database of localized storage data. The data collected by the upper computer is localized and stored in the database through the interface module.

### Overall architecture design

**Figure 3:**
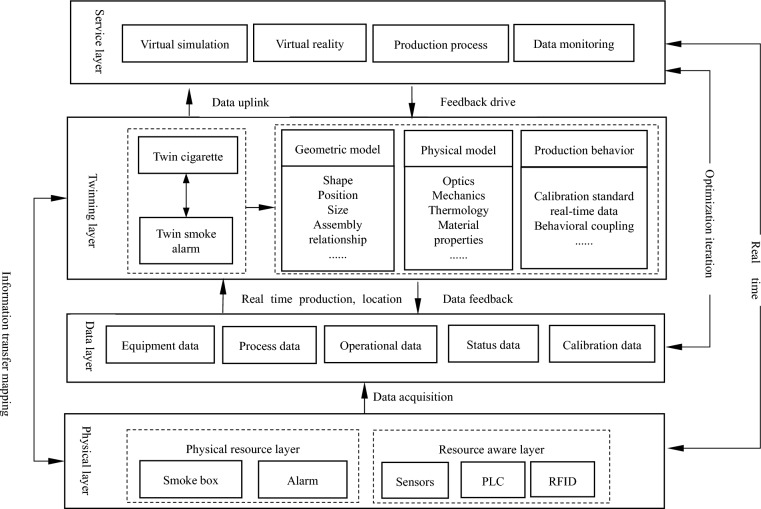
Frame diagram of digital twins.

The application framework of digital twin cigarette box includes physical layer, twin layer, data layer and service application layer. As shown in Fig. 3.

The physical layer includes two layers: physical resource and resource perception. The physical resource layer includes smoke box entity, smoke alarm, etc.; The resource sensing layer includes PLC, optical receiver, flow rate sensor, temperature and humidity sensor, etc.; The physical smoke box entity realizes the real-time perception of the real-time information and working status of the physical smoke box through the data acquisition technologies such as sensors and TCP communication protocol. At the same time, it can upload the real-time data of the physical smoke box collected by the sensors to the virtual smoke box system, and receive the monitoring tasks from the service system to realize the control of the virtual smoke box to the physical smoke box.

The twin layer is the core of the system, and the twin model includes geometric model, physical model and production behavior model^16^. The geometric model is used to express the visualization effect of the physical smoke box mapping in the information space, and has the same shape, size, position parameters and assembly relationship with the physical smoke box; The physical model adds the physical attributes, constraints, characteristics and other information of the physical smoke box on the basis of the geometric model; The production behavior model maps the production operation logic in real time.

The data layer collects data through the acquisition program, and stores the data into the database, including equipment data, process data, operation data, status data, calibration data, etc.

The service application layer realizes virtual simulation, virtual real synchronous simulation, remote control and other functions by accurately mapping the twin layer and data layer. In the virtual simulation mode, the twin cigarette box sets the virtual simulation production process according to the parameters to provide feasibility verification for the actual production; In the virtual real synchronous simulation mode, the physical smoke box status and operation data are updated to its virtual model in real time based on the digital twin mapping mechanism for real-time visual monitoring; Remote control means that based on synchronous simulation and data analysis, the digital twin cigarette box sends instructions to the physical space and reversely controls the physical cigarette box for exception handling.

## Construction of digital twin cigarette box model

**Figure 4:**
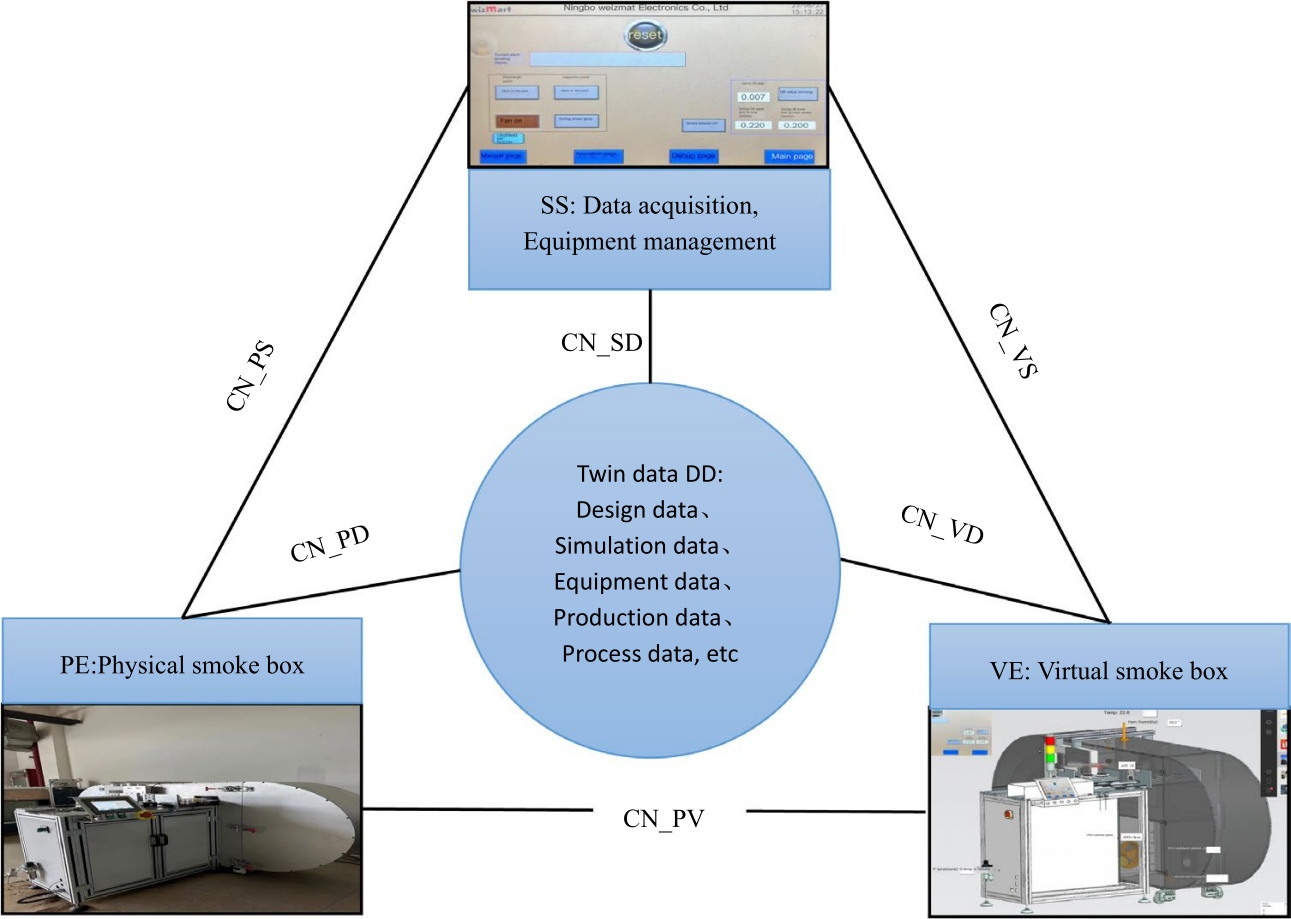
Five dimensional model of digital twin cigarette box.

Combined with the architecture of the digital twin five-dimensional model^17^ and the data integration and system integration capabilities of the industrial Internet of things platform, according to the smoke alarm calibration production situation, a smoke alarm calibration smoke box system model based on the digital twin is constructed. The smoke alarm calibration smoke box system structure is shown in Fig. 4.

It includes physical cigarette box *(PE*), virtual cigarette box (*VE*), service (*SS*), twin data (*DD*) and connection (*CN*), and the digital twin five-dimensional model is shown in Eq. (2).

2 $${M}_{DT}=\left(PE,VE,SS,DD,CN\right)$$

### Physical smoke box

**Figure 5:**
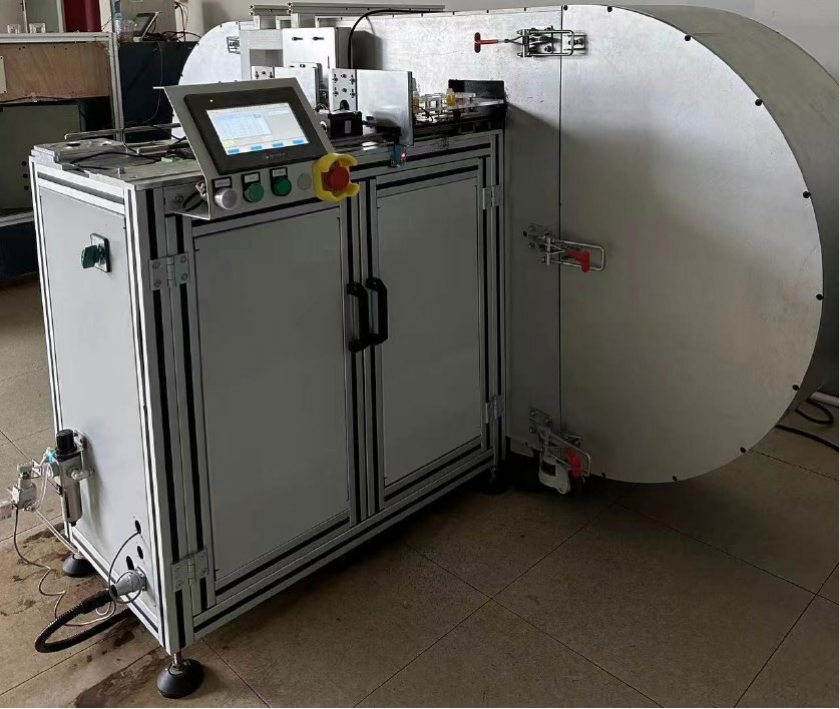
Physical smoke box.

The physical smoke box entity is the foundation of this system. It can be divided into unit level, system level and complex system level by function. Unit level is the smallest manufacturing unit of smoke box, such as box, upper cover, fan, smoke exhaust outlet, air inlet, smoke generator, optical density meter, flow rate sensor, temperature and humidity sensor, etc. The system level is formed by the integration and cooperation of multiple unit levels. The smoke box system level mainly includes mechanical system level, electrical system level, control system level, etc. The complex system level is based on the system level, through the information flow, energy flow and so on to achieve cross system interconnection, interoperability and mutual collaborative optimization. Figure 5 shows the physical smoke box.

### Virtual smoke box

The virtual smoke box includes geometric model (${G}_{v}$), physical model (${P}_{v}$), behavior model (${B}_{v}$) and rule model (${R}_{v}$)^18^. The virtual model describes and depicts the physical entity from multiple time scales and spatial scales, and its mathematical expression is shown in (3).

3 $$\mathrm{VE}=\left({G}_{v}, {P}_{v},{B}_{v},{R}_{v}\right)$$

When building the digital twin cigarette box model, we should first map the size, shape, assembly relationship and other attributes and laws of the physical cigarette box, so that the model can simulate the geometry, constraints and physical attributes of the real physical cigarette box through known experience or physical laws. The second is to express the production behavior of the real cigarette box in the model, which mainly describes the multi-disciplinary, multi physical quantity and multi-scale data information related to the behavior activities, such as mechanical data, electrical data, control data, process data and state data. Finally, through the comparative analysis of the production data of the physical cigarette box and the virtual simulation data, the decision is made according to the relationship and rules between the parameters, and fed back to the real physical cigarette box to realize the monitoring of the production process of the physical cigarette box.

#### Geometric model

**Figure 6:**
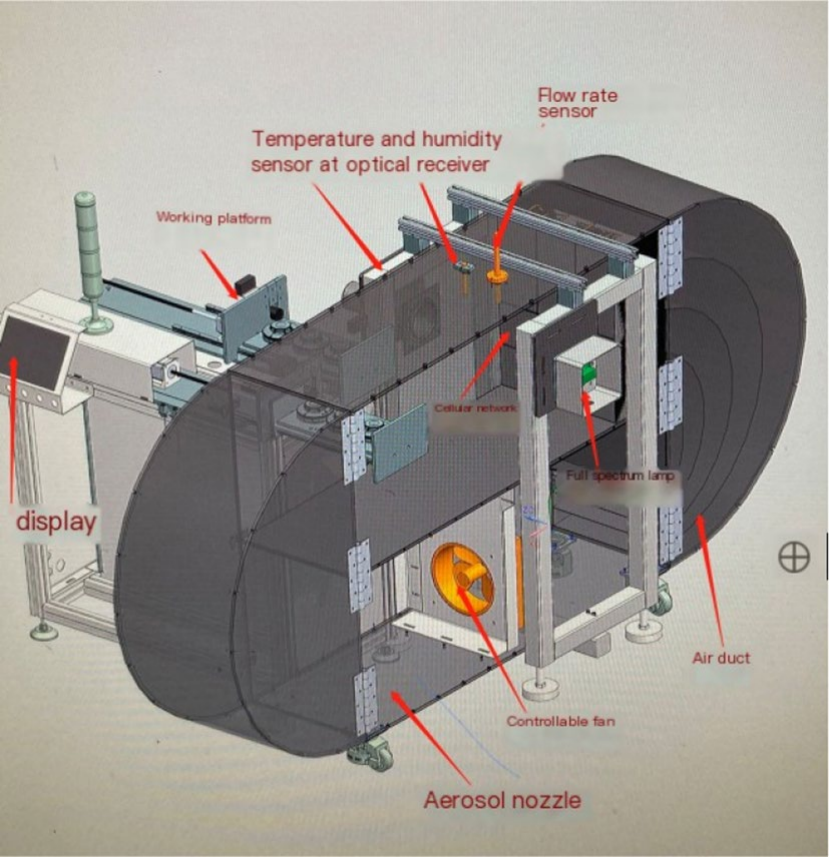
Geometric model of smoke box.

The geometric model is a three-dimensional model that describes the shape, size, position and assembly relationship of the physical entity, and has good temporal and spatial consistency with the physical entity. Figure 6 shows the geometric model of the smoke box. In order to achieve the movement effect of cigarette box calibration, each component must be established according to the assembly relationship. A complete smoke box is usually composed of the main body, moving parts, control system, electrical subsystem, heating device, smoke generation device, speed measuring device, pneumatic subsystem, smoke exhaust system and other ancillary structures. In order to improve the efficiency of computer graphics processing and simulation, the 3D model needs to be lightweight in modeling, and the key components, such as the main body of the smoke box, the working platform, the smoke generating device, the heating device, the alarm, etc., need to be retained; Simplify components that have no obvious relationship with simulation, such as air pressure device, control system, smoke exhaust device, etc., only need to show the complete overall dimensions and accurate motion form of the smoke box, without considering other auxiliary structures. The mathematical language expression for constructing the geometric model is (4):

4 $$G_{v} = (S_{{\text{g}}} ,D_{g} ,P_{g} ,AR_{g} ,PR_{g} )$$

where: *S*_*g*_ is geometric shape, *D*_*g*_ is geometric dimension, *P*_*g*_ is geometric position, *AR*_*g*_ is assembly relationship, *PR*_*g*_ is positional relationship.

#### Physical model

The physical model is to add the physical attributes, constraints, characteristics and other information of the physical smoke box and smoke alarm on the basis of the geometric model. For example, the thermal, mechanical and optical properties of smoke generating device, speed measuring device, temperature measuring device and other structures, the physical properties of the smoke box are realized by adding physical properties such as material, mass, friction, collision body to the geometric model, and the physical model of the smoke box is constructed by adding various sensors to collect the working state of the alarm and the environmental parameters of the smoke box in real time. The mathematical expression of the physical model of the cigarette box is (5):

5 $$P_{v} = (E_{p} ,Y_{p} ,PC,EP,{\text{Info)}}$$

where :*E*_*p*_ is the physical property of the smoke box,* Y*_*p*_ is the physical property of the alarm, *PC* is the physical property of the smoke box, *EP* is the environmental parameter of the smoke box, and Info is other information.

The physical properties of the cigarette box include the model of the cigarette box(*T*_*e*_), the running speed of the cigarette box (*V*_*e*_), the power of the cigarette box (O_*e*_), the light speed wavelength (*L*_*e*_), the optical measurement length(*d*), the radiation power received when smoking(*P*_*0*_), the radiation power received when smoking(P), etc. Its mathematical language expression is (6):

6 $$E_{p} = \left( {T_{e} ,V_{e} ,O_{e} ,L_{e} ,d,P_{0} ,P...} \right)$$

The physical properties of the alarm include the model (*T*_*y*_), status (*S*_*y*_), Working Voltage (*V*_*dc*_), quiescent current(*QC*), alarm current (*AC*), alarm sound pressure(*ASP*), sensitivity (*SS*), etc. Its mathematical language expression is (7):

7 $$Y_{p} = \left( {T_{y} ,S_{y} ,V_{dc} ,{\text{Q}}C{\text{,AC,ASP,SS}}..} \right)$$

Environmental parameters of smoke box include temperature (*T*_*ep*_), humidity (*H*_*ep*_), air pressure (p_*ep*_), wind speed (W_*ep*_), smoke concentration (D_*ep*_), smoke inlet frequency (F_*ep*_), shielding rate (C_*ep*_), etc. Its mathematical language expression is (8):

8 $$EP = \left( {T_{ep} ,H_{ep} ,{\text{p}}_{ep} ,W_{ep} ,D_{ep} ,F_{ep} ,C_{ep} ...} \right)$$

#### Behavior model

Virtual simulation debugging depends on the model construction at the sensor and actuator level, and the behavior model is divided into sensor modeling and actuator modeling. The environmental parameters of the smoke box are set according to the requirements of the calibrated environmental parameters. The corresponding sensors of the smoke box (such as temperature and humidity sensor, flow rate sensor, smoke concentration sensor, etc.) are defined according to the working principle of the sensor. Through the sensor behavior modeling, the digital twin model of the smoke box is fed back to the control system based on the signals generated by the working conditions. In order to increase the accuracy of the trigger calibration time and avoid the error caused by the trigger calibration only by the calculation of the environmental parameters of the smoke box reaching the standard, position sensors are set at the inlet and outlet of each product. The calibration time of the smoke box is determined by calculating the environmental conditions of the smoke box, the trigger outlet sensors of the previous batch of calibrated products and the trigger inlet position sensors of the next batch of products, so as to improve the accuracy and success rate of calibration.

Based on the working principle of the driving smoke box, the motion control parameters of the corresponding actuator are defined, and their interaction signals with the control system are associated with the motion attributes of the digital model. With the help of actuator behavior modeling, the digital model can realize the movement of the actuator driven by the output signal of the control system.

The behavior model data includes process parameter (*P*_*ps*_), process sequence (*P*_*ss*_), smoke box operation data (*R*_*d*_), alarm operation data (*Y*_*rd*_), smoke box environment parameter (*EP*) and other information (*Info*), which are obtained and rendered to the model through the data interface set. Therefore, the mathematical language of cigarette box behavior model is expressed as formula (9):

9 $$B_{v} = \left( {P_{ps} ,P_{ss} ,R_{d} ,Y_{rd}^{{}} ,EP,Info...} \right)$$

#### Rule model

**Figure 7:**
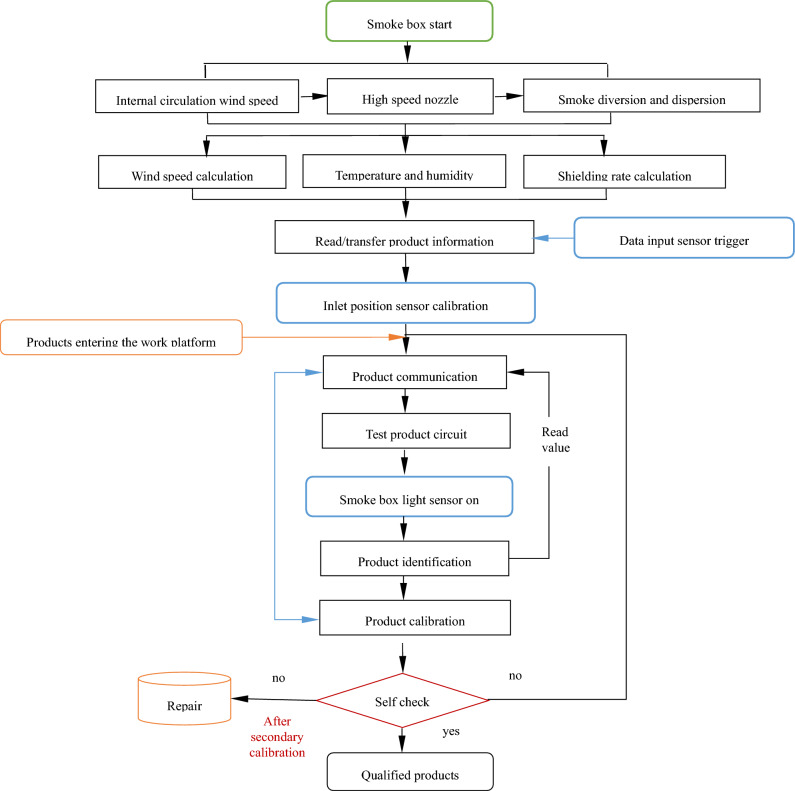
Product calibration process.

The rule model is designed after the behavior model is completed. In order to make the rule model meet the requirements of virtual simulation debugging, it needs to meet the control logic of the system. The calibration process of smoke alarm is shown in Fig. 7. After setting the environmental parameters of the smoke box, the data input sensor triggers to read the product information and write it into the PLC data block to calculate the pulse variable. When the pulse count value in the data block enters the window of the import pulse value, the PLC initiates a calibration request. After the calibration of the import position sensor is completed, the cylinder starts to move, the workbench acts, and the corresponding product starts calibration. After the product is successfully calibrated, the data of the corresponding element in the PLC data block is cleared. If the primary calibration of the product is unsuccessful, the secondary calibration will be carried out. If the calibration of the product is not successful after the secondary calibration, the product will be sent to the repair area.

### Twin data model construction

Data as a bridge connects the physical smoke box and the virtual smoke box. In order to ensure the normal operation of the smoke box in the calibration process, it is necessary to collect data in real time. The virtual cigarette box model maps the physical cigarette box in real time. On the basis of completing the static modeling of the virtual model, the dynamic modeling between the virtual model and the physical entity is required. On the one hand, the physical entity can drive the digital twin model for real-time simulation through the communication system. On the other hand, the digital twin model can also send instructions to reverse control the operation of the physical entity, realizing the two-way mapping of "twins".

#### Communication system architecture

**Figure 8:**
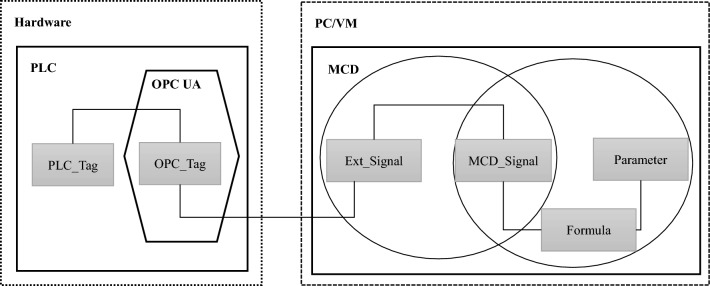
Signal transmission path.

To enable the virtual cigarette box to smoothly map the real-time operation of the physical cigarette box, it is necessary to collect the status data, operation data and location data of the physical cigarette box. The type analysis and format of these data are not unified. The virtual model and external signal connection currently support the following protocols: OPC Da, SHM, OPC UA, TCP, plcsim adv, UDP, PROFINET. The system uses OPC UA communication protocol to build a virtual real mapping platform. The whole environment is composed of three parts: PLC CPU, OPC server and digital twin model (MCD). The CPU is used to process the operation logic, obtain the machine status through the OPC server, and send the operation instructions to the equipment through the OPC server; MCD is used to display the virtual model and conduct simulation, obtain CPU instructions from OPC, and feed back the current equipment status through OPC server. Figure 8 shows the signal transmission path.

#### Data acquisition

**Figure 9:**
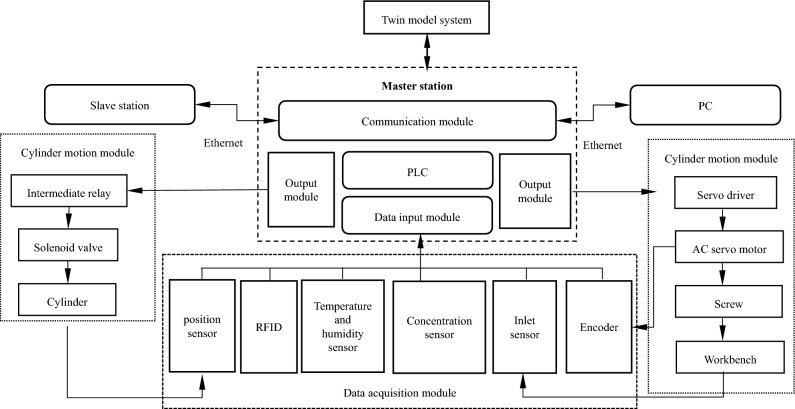
Data acquisition and communication.

Data is the foundation of digital twins, originating from physical entities, twin systems, sensors, etc. It covers twin model data, environmental data, physical entity geometric data, physical data, process data, production and operation data, and runs through the entire process of physical object operation. The realization of data acquisition and communication is shown in Fig. 9. Data acquisition mainly relies on the data acquisition module of the physical cigarette box. The cigarette box is equipped with an RFID reader to automatically read the position, rotation angle, residence time, etc. after the alarm enters the cigarette box; The sensor is used to obtain smoke box working condition data, alarm status data, smoke box environment data, etc. Data information such as position sensor, temperature and humidity sensor and concentration sensor are collected in the calibration system and then input to the master station. The main station stores the received data into real-time data and historical data in the database. Based on the collected information, the control system analyzes the actual situation of each station of the cigarette box, and controls the movement of the cylinder and workbench of each station through the slave station and master station. In addition, the industrial Ethernet communication protocol is used to establish communication between master and slave stations and between master station and host computer (PC), so as to realize the real-time data flow under the digital twin architecture.

The main station provides an interface to the twin model system to drive the operation of the model. All virtual variables generated during the model operation are stored in the database with the same data classification, and then the real workshop variables obtained from the server are traversed to establish a one-to-one connection with the virtual workshop variables, achieving the acquisition of data from the main station by the model.

#### Data mapping and driving

Smoke alarm calibration twin system virtual real mapping and driving is to communicate and connect the equipment status data generated during the actual operation of the physical space of the smoke box with the digital space, and transmit the equipment status data to the digital space twin model to realize the synchronous operation of the physical space smoke box and the digital space twin virtual model. The physical space collects, transmits, analyzes, processes and stores data through software programs, and finally imports it into the database. The data stored in the database is connected to the digital space after unified processing.

**Figure 10:**
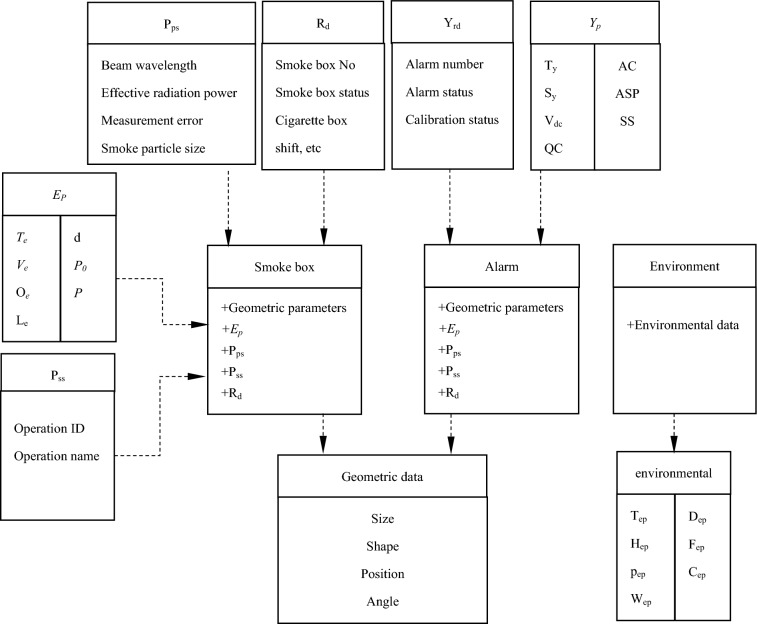
Data subject mapping content.

The data mapping of smoke alarm calibration includes smoke box, alarm, environment mapping, etc. As shown in Fig. 10.

1. Smoke box mapping. The smoke box is the main equipment for calibration production. It carries out real-time mapping of the behavior, position and state of each part of the smoke box in production. The mapping mainly includes the geometric parameters of the smoke box, the physical attribute of the smoke box(*E*_*p*_), the process parameter (P_ps_), the process flow (P_ss_), and the smoke box operation data (R_d_).
2. Alarm mapping. The production data drives the alarm throughout the calibration process, and the data of the alarm in the calibration process includes the geometric data, physical attribute data (*Y*_*p*_), operation data (Y_rd_), etc.
3. Environment mapping. The environmental mapping displays the environmental parameter information in the smoke box in real time during the calibration process, understands the impact of environmental parameter changes on the alarm threshold, and timely adjusts to ensure the accuracy of the threshold. Including: T_ep_, H_ep_, p_ep_, W_ep_, D_ep_, F_ep_, C_ep_, etc.

**Figure 11:**
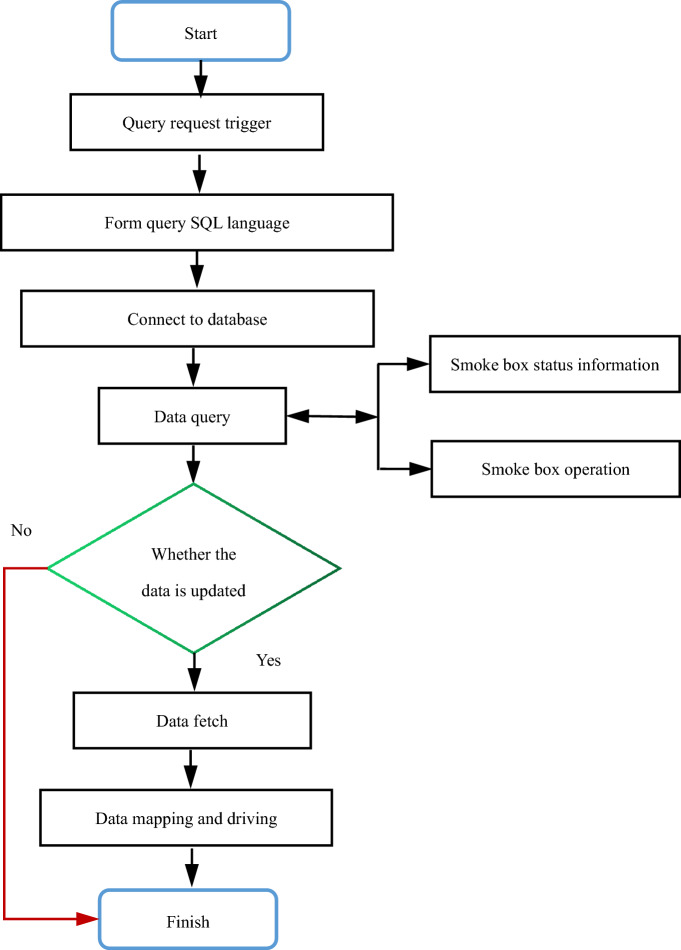
Data driven flowchart.

After getting the operation data of real equipment, we need to use some data to drive the equipment in the digital twin scene to achieve the dynamic consistency between the physical space and the virtual digital twin space. The process of real data driving virtual digital twin is introduced by taking the smoke box monitoring in digital twin system as an example. After receiving the trigger request of data query, the digital twin system for smoke box monitoring circularly reads data from the database at a certain time interval, and then continuously transmits the data to the drive function of the fan equipment. Through continuous real-time rendering and display, the dynamic consistency between the physical world equipment and the twin system equipment is realized. The flow chart is shown in Fig. 11.

First, the user sends a data query request and generates SQL statements according to the corresponding trigger. The system connects the database and queries the corresponding data in the database according to the data request command. After querying the corresponding data, judge the data to determine whether the data in the database has been updated. If the data in the database has been updated, the read data will be written into the model driver function according to the data mapping relationship, and NX will render and display the results in real time. If the data in the database is not updated, end the data acquisition. By periodically cycling the above process, the purpose of real-time data driving is achieved. Complete the corresponding actions of the equipment driven by data.

## Application examples

According to the modeling and mapping method mentioned in this paper, the calibration font of a smoke alarm of a company in Ningbo, Zhejiang Province is used as the verification. According to the calibration process of smoke alarm and the digital twin modeling method proposed in this paper, the Siemens NX platform is used to build the digital twin model of the smoke box, and the OPC UA communication protocol is used to build the virtual real communication platform. Finally, the real-time mapping technology is used to realize the visualization of 3D model and virtual production.

**Figure 12:**
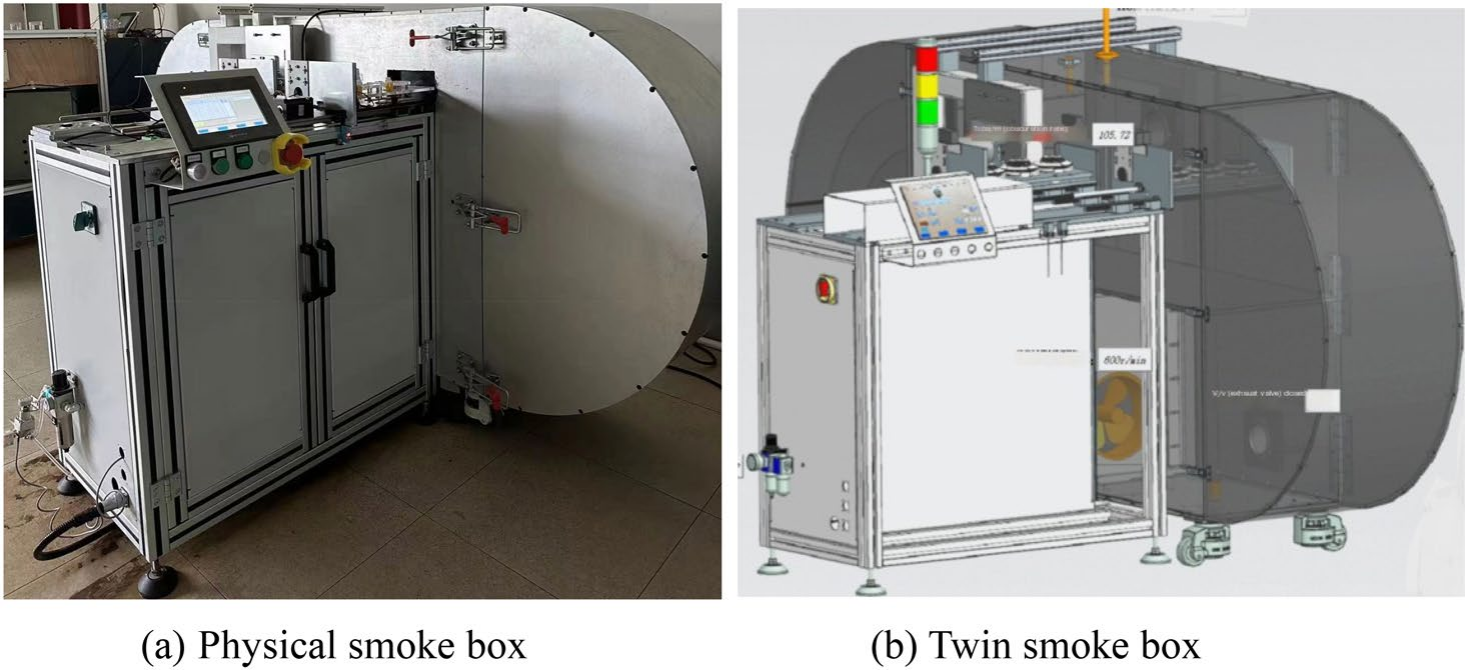
Comparison of physical smoke box and twin smoke box.

**Figure 13:**
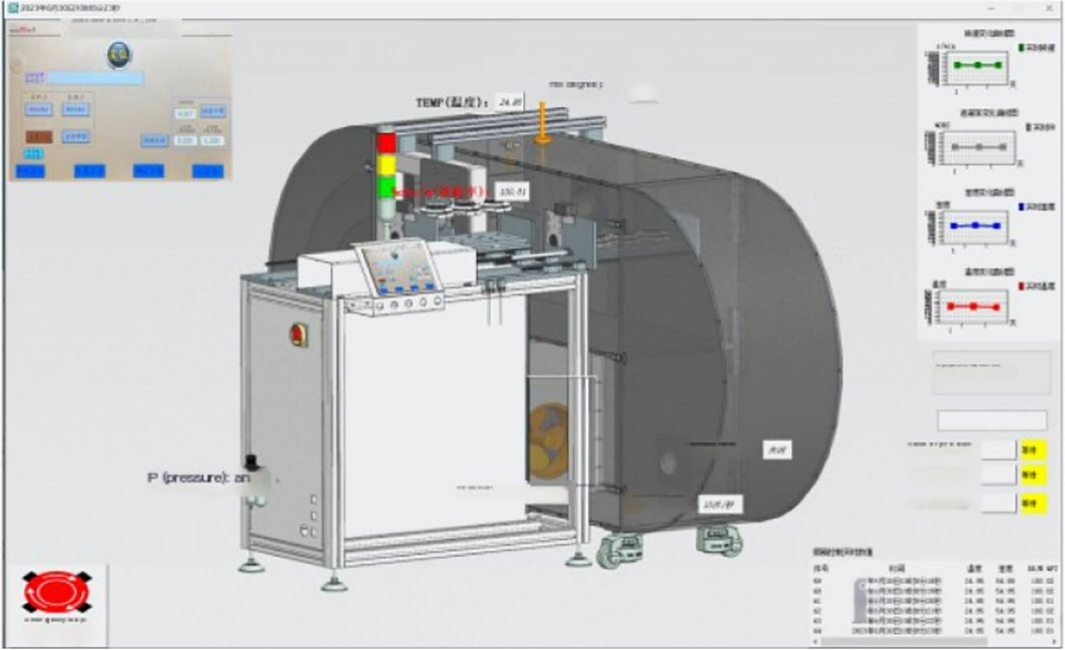
Monitoring control interface during cigarette box production.

**Figure 14:**
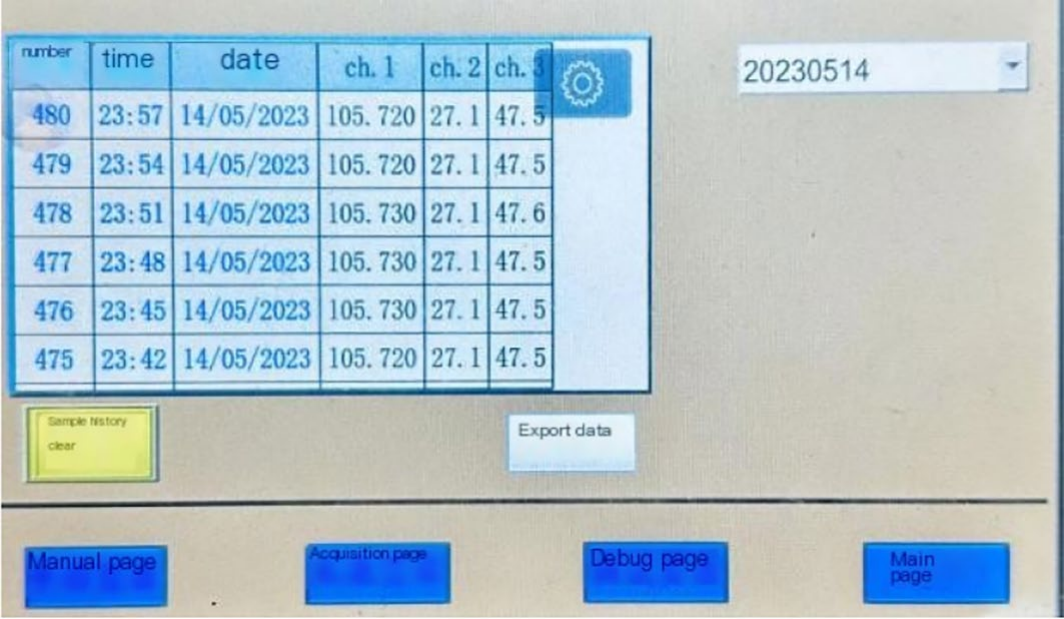
Data acquisition interface.

When building the twin model of the cigarette box, Siemens NX was used to model the whole cigarette box in equal proportion, and the assembly was carried out according to the geometric position relationship and assembly relationship between the components of the cigarette box, and the physical properties such as material, mass, friction, mechanics, optics, and heat corresponding to the model and the physical cigarette box were given to complete the construction of the geometric model and the physical model, as shown in Fig. 12, which shows the comparison between the physical entity of the cigarette box and the twin model. By mapping the position and calibration behavior of the physical smoke box and the alarm, the visual detection of the smoke alarm calibration production process is realized, and the real-time production data is collected and saved in the database. Figure 13 shows the monitoring interface during cigarette box production. Figure 14 data acquisition interface.

**Figure 15:**
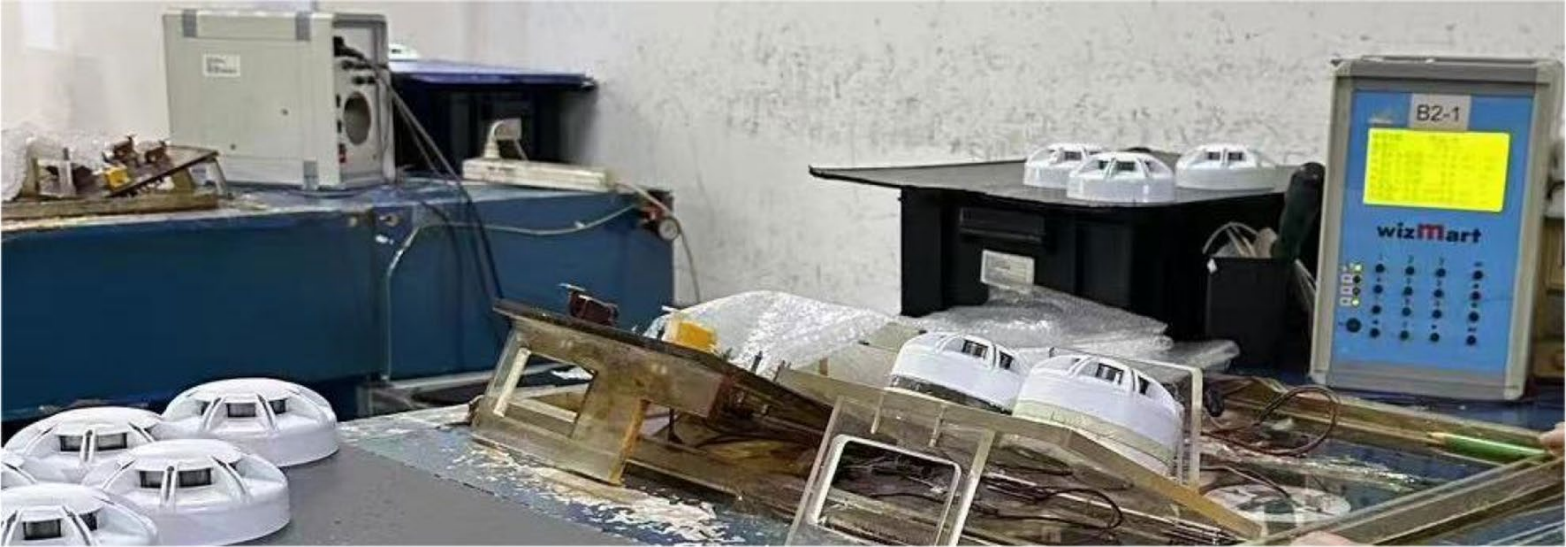
The calibration of traditional smoke alarm.

In order to verify the reliability of the method proposed in the paper, the authors team compared the traditional calibration method with the calibration method proposed in this paper. The traditional calibration method for smoke alarms is to manually place the product into the calibration instrument, observe the calibration instrument data with the naked eye, and then manually collect the product. The operator does not record any calibration data during the calibration process. The traditional smoke alarm calibration method is shown in Fig. 15. The authors randomly selected 40 Rum alarms calibrated using traditional calibration methods for sensitivity testing. Among them, 3 products had sensitivity thresholds that exceeded the standard range. These 40 alarms were recalibrated using the method described in this paper. After testing, the sensitivity of all products met the requirements of EN54-S/7:2000 testing criteria, as shown in Table 1. Through multiple inspections and verifications, compared with traditional calibration methods, the qualification rate of this design system has increased from 98 to 99.6%, and the repair rate of defective products has decreased by 1.6%.

**Table 1:** Sensitivity test report.

Product name:Rum90		Test method :EN54-S/7:2000	
Alarm number	Alarm value		Unit
	The traditional method	The proposed method	
1	0.155	0.155	db/m %FT
2	0.130	0.130	db/m %FT
3	0.160 (unqualified)	0.154	db/m %FT
4	0.120	0.120	db/m %FT
5	0.126	0.126	db/m %FT
6	0.139	0.139	db/m %FT
7	0.145	0.145	db/m %FT
8	0.124	0.124	db/m %FT
9	0.131	0.131	db/m %FT
10	0.126	0.126	db/m %FT
11	0.119	0.119	db/m %FT
12	0.122	0.122	db/m %FT
13	0.124	0.124	db/m %FT
14	0.119	0.119	db/m %FT
15	0.153	0.153	db/m %FT
16	0.114	0.114	db/m %FT
17	0.131	0.131	db/m %FT
18	0.142	0.142	db/m %FT
19	0.135	0.135	db/m %FT
20	0.117	0.117	db/m %FT
21	0.119	0.119	db/m %FT
22	0.160 (unqualified)	0.145	db/m %FT
23	0.127	0.127	db/m %FT
24	0.136	0.136	db/m %FT
25	0.119	0.119	db/m %FT
26	0.126	0.126	db/m %FT
27	0.126	0.126	db/m %FT
28	0.145	0.145	db/m %FT
29	0.135	0.135	db/m %FT
30	0.117	0.117	db/m %FT
31	0.124	0.124	db/m %FT
32	0.127	0.127	db/m %FT
33	0.147	0.147	db/m %FT
34	0.124	0.124	db/m %FT
35	0.127	0.127	db/m %FT
36	0.147	0.147	db/m %FT
37	0.124	0.124	db/m %FT
38	0.140	0.140	db/m %FT
39	0.157(unqualified)	0.150	db/m %FT
40	0.129	0.129	db/m %FT
Judgment criteria		0.085–0.155	db/m %FT

## Conclusion

This paper preliminarily explores the application of digital twin technology in the smoke alarm calibration workshop, expounds the working mechanism of the smoke alarm calibration system, designs the overall framework of the digital twin smoke alarm calibration and monitoring system, uses Siemens NX platform to build the digital twin five-dimensional model of the smoke box, and uses OPC UA communication protocol to build the virtual and real communication platform RFID and other technologies collect data from the physical smoke box and the twin smoke box, realize the real-time mapping between the physical smoke box and the twin smoke box, improve the efficiency and quality of smoke alarm calibration, and provide a new idea for the digital production of smoke alarm.

## Data Availability

The data used to support the findings of this study are included within the article. The datasets used and/or analyzed during the current study are included in this published article and can be available from the corresponding author on reasonable request.
